# Pancreatic Cancer with Liver Oligometastases—Different Patterns of Disease Progression May Suggest Benefits of Surgical Resection

**DOI:** 10.3390/jcm14155538

**Published:** 2025-08-06

**Authors:** Nedaa Mahamid, Arielle Jacover, Angam Zabeda, Tamar Beller, Havi Murad, Yoav Elizur, Ron Pery, Rony Eshkenazy, Talia Golan, Ido Nachmany, Niv Pencovich

**Affiliations:** 1 Department of General Surgery and Transplantation, Sheba Medical Center, Tel-Hashomer, Gray Faculty of Medicine and Health Sciences, Tel-Aviv University, Tel-Aviv 69978, Israel; neda2.94@gmail.com (N.M.); arielle.jacover@mail.huji.ac.il (A.J.); zabedaangam@gmail.com (A.Z.); ron.pery@sheba.health.gov.il (R.P.); rony.eshkenazy@sheba.health.gov.il (R.E.); ido.nachmany@sheba.health.gov.il (I.N.); 2 Institue of Oncology, Sheba Medical Center, Tel-Hashomer, Gray Faculty of Medicine and Health Sciences, Tel-Aviv University, Tel-Aviv 69978, Israel; tamar.beller@sheba.health.gov.il (T.B.); talia.golan@sheba.health.gov.il (T.G.); 3 Biostatistics and Biomathematics, Data and Analytics Division, Sheba Medical Center, Tel-Hashomer, Gray Faculty of Medicine and Health Sciences, Tel-Aviv University, Tel-Aviv 69978, Israel; havim@gertner.health.gov.il; 4 Department of Internal Medicine, Ward ‘B’, Sheba Medical Center, Tel-Hashomer, Gray Faculty of Medicine and Health Sciences, Tel-Aviv University, Tel-Aviv 69978, Israel; yoav.elizur@sheba.health.gov.il

**Keywords:** chemotherapy, adenocarcinoma, FOLFIRINOX, metastasis, systemic disease

## Abstract

**Background:** Pancreatic adenocarcinoma (PDAC) with liver oligometastases (LOM) presents a therapeutic challenge, with optimal management strategies remaining uncertain. This study evaluates the long-term outcomes, patterns of disease progression, and potential factors influencing prognosis in this patient subset. **Methods:** Patients diagnosed with PDAC and LOM were retrospectively analyzed. Disease progression patterns, causes of death, and predictors of long-term outcomes were assessed. **Results:** Among 1442 patients diagnosed with metastatic PDAC between November 2009 and July 2024, 129 (9%) presented with LOM, defined as ≤3 liver lesions each measuring <2 cm. Patients with LOM had significantly improved overall survival (OS) compared to those with high-burden disease (*p* = 0.026). The cause of death (local regional disease vs. systemic disease) could be determined in 74 patients (57%), among whom age at diagnosis, history of smoking, and white blood cell (WBC) count differed significantly between groups. However, no significant difference in OS was observed between the two groups (*p* = 0.64). Sixteen patients (22%) died from local complications of the primary tumor, including 6 patients (7%) who showed no evidence of new or progressive metastases. In competing risk and multivariable analysis, a history of smoking remained the only factor significantly associated with death due to local complications. **Conclusions:** Approximately one in five patients with PDAC-LOM died from local tumor-related complications—some without metastatic progression—highlighting a potential role for surgical intervention. Further multicenter studies are warranted to refine diagnostic criteria and better identify patients who may benefit from surgery.

## 1. Introduction

Pancreatic ductal adenocarcinoma (PDAC) remains one of the most lethal malignancies, with a dismal prognosis largely due to late-stage diagnosis and aggressive biological behavior [1]. At presentation, more than 50% of patients already exhibit metastatic disease, most commonly to the liver [2,3]. Among those with non-metastatic disease, resectability is determined by anatomical, biological, and functional criteria, classifying tumors as resectable, borderline resectable, or locally advanced [4,5]. Surgical resection of a localized PDAC remains the only potentially curative option, and favorable outcomes have been observed in patients who receive neoadjuvant chemotherapy, followed by surgery, and subsequent adjuvant therapy. However, only a minority of patients are eligible for surgery due to vascular involvement or distant metastases [2,6].

Historically, even a single distant metastasis in PDAC was viewed as the “tip of the iceberg”—an indicator of widespread systemic dissemination that dictated a poor prognosis. As a result, surgical resection of the primary tumor was considered futile, and patients with metastatic disease were managed primarily with systemic chemotherapy or palliative care [7,8]. However, the introduction of more effective systemic chemotherapy regimens—most notably FOLFIRINOX—has resulted in improved disease control and survival in selected patients with advanced PDAC [9]. These advances have prompted interest in surgical resection for patients with low-volume metastatic disease, either performed concurrently with or following resection of the primary tumor. This evolving paradigm challenges long-standing treatment dogma and remains controversial, supported thus far by limited but growing clinical evidence [10]. Liver oligometastatic disease (LOM) is most commonly defined as three or fewer liver-only metastases without extrahepatic spread. Several studies have reported improved long-term outcomes with surgical intervention compared to non-surgical palliative approaches in patients with LOM [11,12,13,14]. Moreover, favorable tumor biology, as well as additional factors, such as a low CA 19-9 level, good performance status, and a response to therapy [15,16,17], were investigated to better identify patients most likely to benefit from radical resection of both the primary tumor and metastases [7,18]. Notably, a proportion of patients with metastatic PDAC ultimately succumb to local complications of the primary tumor, such as biliary obstruction, bleeding, or bowel obstruction [19]. We recently proposed that selected patients with PDAC-LOM may benefit from resection of the primary tumor, regardless of metastatic status [20]. In this study, we examine a cohort of patients with PDAC-LOM to evaluate patterns of disease progression and causes of death. Identifying predictors of progression and mortality may guide surgical decision-making for this unique patient population.

## 2. Materials and Methods

**Figure 1:**
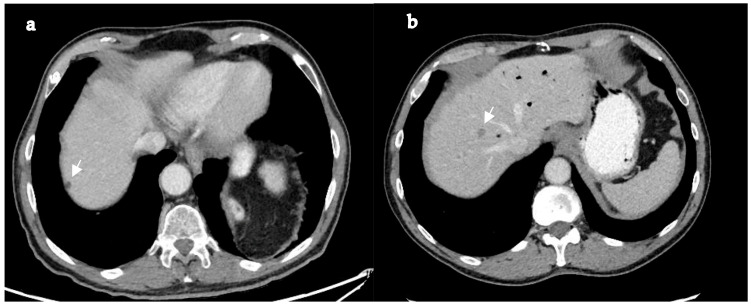
Representative axial contrast-enhanced CT images of a patient with PDAC-LOM. (**a**) Arrows hypoattenuating lesion in liver segment VII measuring 8 mm, consistent with a liver metastasis. (**b**) Arrows hypoattenuating lesion in liver segment VIII measuring 7 mm.

This retrospective study included all patients with metastatic PDAC treated at a tertiary referral center between November 2009 and July 2024. Patients were classified as having PDAC-LOM if they had no more than three liver lesions on imaging that were highly suggestive of PDAC metastases, with each lesion measuring less than 2 cm in maximal diameter, and no evidence of extrahepatic disease [16]. Lesions were determined to be pancreatic metastases based on imaging findings. Cross-sectional imaging was reviewed by a senior radiologist specializing in hepatobiliary and pancreatic imaging. In cases where the primary tumor was FDG-avid, positron emission tomography (PET) was used to support the diagnosis. An example of LOM is shown in Figure 1. All cases were reviewed in multidisciplinary team (MDT) meetings that included pancreatic cancer oncologists, radiation oncologists, radiologists, hepatopancreatobiliary surgeons, upper gastrointestinal surgeons, and gastroenterologists. Data were retrospectively extracted from electronic medical records maintained by the surgery and oncology departments using MDClone© software, a data extraction and synthesis platform integrated with the institution’s records system (MDClone, Beer Sheva, Israel, http://www.mdclone.com).

Collected data included patient demographics, medical history, comorbidities, laboratory values, tumor characteristics on imaging, details of systemic therapy, disease progression, and survival outcomes, including cause of death. All extracted data were manually reviewed and validated. Non-coded data, such as MDT decisions and clinical details, including the specific cause of death, were manually retrieved from patient charts. The cause of death was determined based on a thorough review of the medical records. All study procedures were conducted in accordance with the Declaration of Helsinki. The study was approved by the institutional review board of Sheba Medical Center (approval reference number SMC-9498-22), and the requirement for informed consent was waived due to the retrospective nature of the study.

### Statistical Analysis

Differences between groups were assessed using the Mann–Whitney U rank-sum test for continuous variables and either Fisher’s exact test or the chi-squared test for categorical variables. Patients with missing data were excluded from the calculation of medians, percentages, and statistical comparisons for the respective variable. A two-sided *p*-value ≤ 0.05 was considered statistically significant. Survival analysis was performed using Cox proportional hazards regression, and statistical significance between Kaplan–Meier survival curves was assessed using the log-rank test. To account for the two competing risks—death due to local complications and death due to systemic disease—we employed the Fine and Gray subdistribution hazard model [21] in two separate analyses, using the PHREG procedure [22]. In the first analysis, the event of interest was death due to local complications, with death from systemic disease treated as the competing risk. In the second, these roles were reversed. Patients (*n* = 46) whose cause of death could not be determined were censored using a cause-specific approach, under the assumption of non-informative censoring (i.e., the reason for the unknown cause of death is unrelated to the risk of either event type). Subdistribution hazard ratios for each covariate were presented in a forest plot. Where applicable, cumulative incidence functions were displayed graphically. Multivariable models for both event types were constructed based on covariates identified in the univariate forest plots, treating the alternative cause of death as a competing risk. All statistical analyses were performed using SAS software (version 9.4 for UNIX) and RStudio (version 3.6.2).

## 3. Results

Among 1442 patients diagnosed with metastatic PDAC between November 2009 and July 2024, 129 (9%) presented with LOM, defined as ≤3 liver lesions, each measuring less than 2 cm. Of these, 9 patients (7%) were still alive at data retraction.

**Figure 2:**
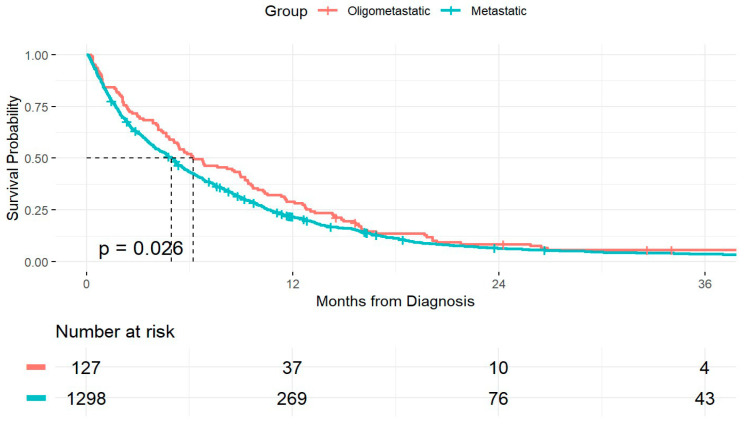
Kaplan–Meier survival curve comparing OS in patients with PDAC-LOM to those presenting with widespread metastatic disease. Median OS is indicated by dashed lines.

When comparing OS between patients who met criteria for LOM and those with other forms of metastatic PDAC at presentation, patients with LOM had significantly improved OS (*p* = 0.026). Median OS was also significantly longer in the LOM group, at 6.2 months (95% CI: 5.09–9.23), compared to 4.9 months (95% CI: 4.53–5.29) in patients with widespread metastatic disease (Figure 2). Based on these findings, we decided to conduct a deeper analysis of the LOM-specific subgroup. Among the 129 patients with PDAC-LOM, the cause of death could be specifically determined in 74 patients (57.3%). Sixteen patients (21.6%) died from local complications of the primary tumor, and among them, six patients (7%) had no evidence of new metastatic lesions. Systemic disease progression with widespread metastases was identified as the cause of death in 58 patients (78.4%). Demographic and clinical characteristics of PDAC-LOM patients, stratified by cause of death, are presented in Table 1. Patients with an undetermined cause of death were excluded from the comparative analysis. Patients who died from local complications of the primary tumor were older (*p* = 0.02), had higher rates of smoking history (*p* = 0.01), and presented with lower WBC counts (*p* < 0.01) compared to those who died from systemic disease (Table 1). Of note, the platelet-to-lymphocyte ratio (PLR) and neutrophil-to-lymphocyte ratio (NLR) were comparable between the groups (Table 1).

We further analyzed patterns of disease progression in patients with LOM. Of the total cohort of 129 patients, 42 patients (32%) had incomplete follow-up data and were excluded from this analysis. Among the remaining 87 patients, 6 (7%) showed no progression of metastatic disease. The majority—41 patients (47%)—experienced progression involving liver and/or distant sites. Among them, 13 patients (32%) had progression of known liver metastases along with new non-liver metastases, but no new liver lesions. 28 patients (68%) developed new liver lesions in addition to new extrahepatic metastases. Five patients from the entire cohort (6%) developed new non-liver metastases only, without progression of the disease in the liver. Progression confined to the liver only was observed in 35 patients (40%). Within this subgroup, 22 (63%) demonstrated both growth of the original LOM and new liver lesions. An additional 7 (20%) had growth of the original liver metastases only, and 6 (17%) developed new liver lesions without progression of the original LOM. These patterns are summarized in Table 2. Notably, a “replaced liver” pattern was identified in 7 patients (8%) of the overall cohort of 87 patients.

**Figure 3:**
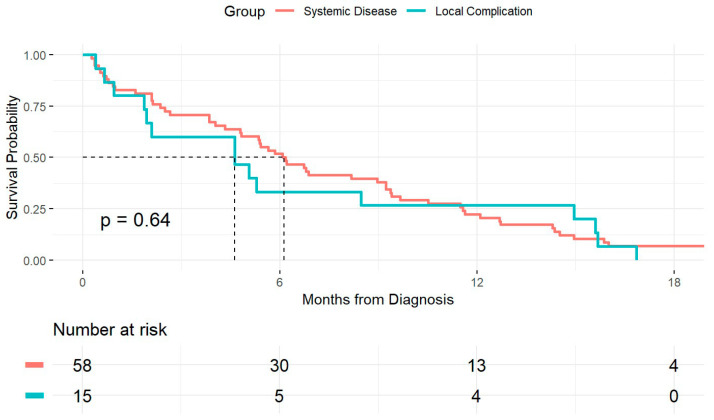
Kaplan–Meier survival curve comparing OS in patients with PDAC-LOM according to the cause of death. Patients who died from local complications are compared to those who died from systemic disease. Median OS is indicated by dashed lines.

**Figure 4:**
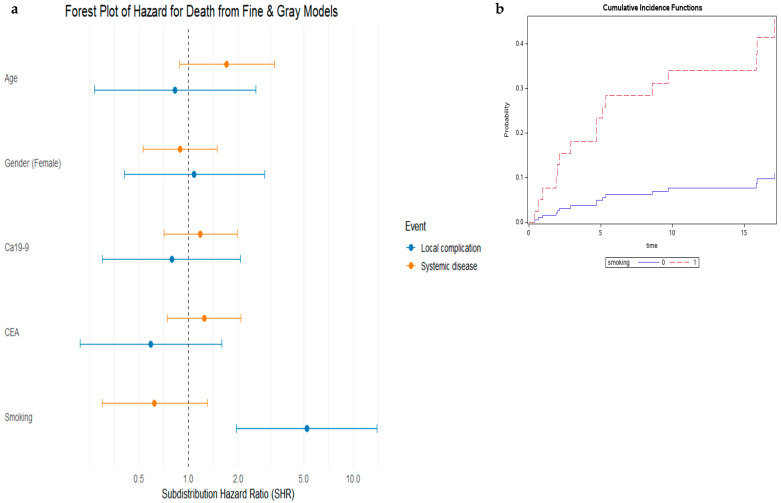
(**a**). Forest plot showing the subdistribution hazard ratios (SHRs) for death due to local complications (blue) and systemic disease (orange) in patients with PDAC-LOM analyzed using Fine and Gray competing risks models. The plot illustrates the association of clinical variables—age at diagnosis (≥65 years), gender (female), CA19-9 (≥500 U/mL), elevated CEA, and history of smoking—with the subdistribution hazard for each event type. Error bars represent 95% confidence intervals. An SHR greater than 1 indicates an increased hazard, while an SHR less than 1 suggests a reduced hazard for the respective cause of death. Active smoking was significantly associated with a higher risk of death from local complications. (**b**). Cumulative incidence functions illustrating the probability of death from local tumor-related complications over time, stratified by smoking status and accounting for the competing risk of death from systemic disease progression. Smokers are represented by a dashed red line, and non-smokers by a solid blue line. The plot demonstrates a higher cumulative incidence of death from local complications among smokers compared to non-smokers.

We subsequently compared patients who died from local complications of the primary tumor to those who died from disseminated metastatic disease. Of the 129 patients in the cohort, excluding the 9 who were alive at the time of data collection, the cause of death was identifiable in 74 patients. Among these, 16 (22%) died from local complications, while 58 (78%) died from disseminated disease. Patients who died from local complications or systemic disease had comparable OS (*p* = 0.64). The median OS was shorter in those who died from local complications, at 4.63 months (95% CI: 1.94–15.6), compared to 6.13 months (95% CI: 4.83–9.23) in those who died from systemic disease; however, this difference was not statistically significant (Figure 3). We further assessed baseline factors that could be associated with the cause of death in patients with PDAC-LOM. Using a competing risk analysis with the Fine and Gray subdistribution hazard model, we evaluated age, smoking history, gender, CA19-9, and CEA levels for their association with cause-specific mortality. As shown in the univariate analysis (Figure 4a), a history of smoking was associated with a significantly increased subdistribution hazard for death due to local complications compared to non-smokers (HR = 5.21, 95% CI: 1.96–13.89; *p* = 0.001). Age ≥ 65 years showed a borderline association with death from systemic disease (HR = 1.70, 95% CI: 0.88–3.31; *p* = 0.11). Other clinical variables, including gender, CA19-9, and CEA levels, were not significantly associated with either cause of death. These findings highlight smoking as a potential risk factor for death due to local tumor-related complications in patients with PDAC-LOM (Figure 4b).

In a multivariable survival analysis, which accounted for competing risks, a history of smoking remained the only factor significantly associated with death due to local complications of the primary tumor in patients with PDAC-LOM (HR = 5.08, 95% CI: 1.93–13.39; *p* = 0.001). No significant association was observed between smoking and death due to systemic disease (HR = 0.62, 95% CI: 0.30–1.92; *p* = 0.20). Other clinical variables, including age > 65, gender, CA19-9 > 500, and elevated CEA levels, were not significantly associated with either local or systemic causes of death in the adjusted model (Table 3).

## 4. Discussion

Although metastatic PDAC is associated with a dismal prognosis, efforts have been made to identify subsets of patients who may benefit from surgical interventions beyond systemic therapy [9,12,18]. Several studies have demonstrated that patients with PDAC-LOM may benefit from radical surgical interventions [13,14,18]. However, careful patient selection is crucial to minimize futile surgeries. Moreover, current approaches typically advocate for resection of both the primary tumor and liver metastases, which may necessitate major hepatectomy and impose a substantial surgical burden [14]. We previously argued that there are essentially two clinical scenarios in patients with PDAC-LOM based on the notion that metastases do not metastasize. In the more common scenario, the liver oligometastases represent the “tip of the iceberg,” with imminent widespread dissemination, rendering resection of both the primary tumor and metastases futile. In the less frequent but clinically relevant scenario, the disease displays relatively “favorable” biology, where full-blown metastasis is not imminent, and existing liver lesions are unlikely to cause short-term complications. In such cases, resecting only the primary tumor may offer benefit [8,23,24]. In this study, we evaluated the natural history of patients with PDAC-LOM who did not undergo surgery, focusing on patterns of disease progression and cause of death, specifically whether death was due to local complications of the primary tumor or systemic disease progression. 

First, we found that patients with PDAC-LOM had significantly improved OS compared to patients with polymetastatic PDAC, supporting further investigation of this distinct subgroup. Second, approximately 22% of PDAC-LOM patients died from complications of the primary tumor, and in some cases, this occurred without the development of new metastases. Third, those who died from local complications were older, had higher rates of smoking history, and had lower WBC counts. Notably, a history of smoking was independently associated with increased risk of death from local complications.

The finding that roughly one in five patients died from local tumor-related complications suggests that selected patients may benefit from surgery addressing the primary lesion. Although pancreaticoduodenectomy at our center—as in other high-volume hepatopancreatobiliary centers—carries a significant risk of morbidity (Clavien–Dindo grade III or higher in approximately 20% of patients) and a 90-day mortality rate of 3%, it may prevent life-threatening local complications such as cholangitis, gastrointestinal bleeding, or bowel obstruction in appropriately selected individuals [25].

Moreover, procedures with a more favorable complication profile—such as bilio-enteric or gastro-enteric bypass (or both, in the case of a double bypass)—may be sufficient to relieve biliary or gastric outlet obstruction. However, these less invasive interventions are unlikely to prevent hemorrhagic complications [26,27].

Importantly, OS among patients who died from local complications was comparable to those who died from systemic progression, suggesting potential biological differences between these subgroups. In fact, 7% of patients died from local complications without any evidence of metastatic progression, raising the possibility that resection of the primary tumor may have meaningfully prolonged survival in these cases.

However, any potential benefit must be weighed against the substantial morbidity and possible delays in systemic therapy associated with pancreatic surgery. Postoperative complications can impair performance status and may limit the ability to resume oncologic treatment in a timely manner, an especially relevant concern in patients with metastatic disease. Although our study does not include patients who underwent resection, these findings underscore the need for prospective studies evaluating whether surgical interventions aimed at local disease control could improve outcomes in highly selected patients with PDAC-LOM.

A major challenge remains: identifying at presentation which patients are likely to die from local complications. Among the clinical variables assessed, smoking history emerged as the most prominent factor associated with local-complication-related mortality. Prior studies have established that smoking significantly increases both the incidence and progression of PDAC. Smokers have a twofold higher risk of developing PDAC, with risk increasing alongside smoking intensity and duration [28,29]. Smoking also promotes PDAC cell invasion and metastasis via multiple molecular mechanisms [30,31]. Interestingly, a study by Kruger et al. showed that non-smoking females had a higher rate of metastatic PDAC compared to smoking males [32]. Here, we show for the first time that smoking is not only associated with PDAC development and spread but also with distinct patterns of disease progression. Specifically, patients with a current or past smoking history were significantly more likely to die from complications of the primary tumor than from systemic disease progression.

Patients who died from local complications were also, on average, five years older than those who died from systemic disease. This finding is consistent with previous data suggesting that younger patients are more likely to present with metastatic disease [33,34]. We also observed that patients who died from local complications had lower WBC counts than those who died from systemic progression. While this finding is statistically significant, the WBC values in both groups remained within normal clinical ranges, limiting their practical relevance. Moreover, PLR and NLR, which have previously been shown to have some prognostic value in patients with PDAC [35], were comparable between the groups.

While clinical decision-making for surgery in PDAC-LOM cannot rely solely on age or smoking status, our findings suggest that distinct predictors of disease progression patterns do exist. In the era of modern systemic therapies such as (m)FOLFIRINOX, a minority of patients may exhibit an exceptional clinical or biochemical response, potentially making them candidates for local therapy, including surgery [36]. However, PDAC-LOM remains primarily a systemic disease, and surgery should only be considered as an adjunct in patients with demonstrated control of systemic progression. With a larger cohort, future studies may help identify more refined and clinically actionable biomarkers that can guide selection for multimodal treatment strategies in this challenging patient population.

This study has several important limitations. First, its retrospective design may introduce selection bias and limit the ability to infer causality between clinical characteristics and outcomes. Second, the determination of the cause of death was based on clinical judgment and chart review, which, despite rigorous validation, may be prone to misclassification. Third, the relatively small sample size of patients with PDAC-LOM, particularly those who died from local complications, limits the generalizability of our findings and the statistical power of subgroup comparisons. Fourth, this study did not identify the subgroup of patients who experienced rapid progression under systemic therapy. Given the significant morbidity associated with primary tumor resection, such an approach should be pursued only after a documented response—or at least the absence of early progression—on systemic treatment. These findings support the potential role of multimodal therapy, combining systemic and local treatment, in carefully selected patients. Finally, while we identified smoking as a potential predictor of disease progression pattern, other relevant biological or molecular markers were not assessed and may provide additional insights in future studies.

## 5. Conclusions

A subset of patients with PDAC-LOM experience death from local tumor-related complications rather than from systemic disease progression. These patients had OS comparable to those who died from metastatic spread, suggesting potentially meaningful differences in underlying tumor biology. Smoking history emerged as a significant predictor of death due to local complications, highlighting its possible role in shaping disease trajectory. These findings support the concept that careful patient selection based on clinical and biological factors may help identify candidates for multimodal therapy, including surgical intervention, aimed at mitigating local disease burden. Prospective studies with larger cohorts are needed to refine risk stratification and guide personalized, multimodal treatment strategies in this challenging patient population.

## Figures and Tables

**Table 1:** Characteristics of patients with PDAC-LOM according to the cause of death.

Characteristic ^1^	Total PDAC-LOM Patients with Known Cause of Death *n* = 74	Death from Local Complications *n* = 16	Death from Systemic Disease *n* = 58	*p*-Value ^2^
Age at diagnosis	73 (10.7)	77.5 (23)	72 (8)	0.02
Female gender	30 (39%)	7 (44%)	23 (40%)	0.99
BMI (kg/m^2^)	24 (4)	24 (3.5)	24	0.69
Smoking history	14 (18%)	7 (43%)	7 (12%)	0.01
Diabetes	23 (29%)	5 (31%)	18 (31%)	>0.9
HTN	36 (46%)	9 (56%)	27 (46%)	0.68
CVA	4 (5%)	1 (6%)	3 (5%)	>0.9
CA19-9	788 (4528)	392 (2204.75)	825 (6777)	0.79
CEA	5 (14.1)	3.35 (4.5)	6.5 (17.8)	0.16
Bilirubin (mg/dL)	0.6 (2.5)	1 (5.8)	0.6 (1.98)	0.34
Albumin (g/dL)	4 (0.3)	3.9 (1)	4 (0.3)	0.25
Creatinine (mg/dL)	0.7 (0.3)	0.75 (0.34)	0.7 (0.2)	0.28
WBC (K/microL)	7 (3)	5.5 (3.25)	7 (3)	<0.01
PLR (K/microL)	130.56 (108.5)	115 (76)	141.4 (102)	0.37
NLR (K/microL)	2.75 (1.8)	3.12 (1.58)	2.64 (1.84)	0.9

^1^ Median (IQR); *n* (%), ^2^ Mann–Whitney U test; Pearson’s chi-squared test; Fisher’s exact test. BMI—body mass index, HTN—hypertension, CVA—cerebrovascular accident, CEA—carcinoembryonic antigen, WBC—white blood cells, PLR—platelet to lymphocyte ratio, NLR—neutrophil to lymphocyte ratio.

**Table 2:** Patterns of disease progression in patients with PDAC-LOM.

Characteristic	Total ^1^
No progression of metastases	6 (7%)
Progression of liver disease and new disseminated metastases ^2^	41 (47%)
• Progression of known LOM + new non-liver metastases	13 (32%)
• New liver lesions + new non-liver metastases	28 (68%)
New non-liver lesions only	5 (6%)
Progression of liver disease only	35 (40%)
• Growing of the original LOM + new lesions	22 (63%)
• Growing of the original LOM only	7 (20%)
• New liver lesions only	6 (17%)

^1^ *n* (%) out of 87. ^2^ lung, peritoneal, and retroperitoneal metastases.

**Table 3:** Multivariable analysis of predictors for cause of death.

Variable	Multivariable Analysis— Local Complication		Multivariable Analysis— Systemic Disease	
	Hazard Ratio 95% CI	*p*-Value	Hazard Ratio 95% CI	*p*-Value
Age	0.89 (0.31–2.56)	0.83	1.68 (0.84–3.39)	0.14
Female gender	1.24 (0.46–3.30)	0.67	0.81 (0.46–1.44)	0.47
Smoking history	5.08 (1.93–13.39)	0.001	0.62 (0.30–1.29)	0.21
CA19-9	1.06 (0.31–3.66)	0.92	1.05 (0.56–1.97)	0.88
CEA	0.62 (0.18–2.14)	0.45	1.09 (0.58–2.04)	0.79

## Data Availability

The data presented in this study are available on request from the corresponding author due to privacy and ethical restrictions.

## Abbreviations

The following abbreviations are used in this manuscript:

PDAC	pancreatic adenocarcinoma
LOM	liver oligometastases
MDT	multidisciplinary team
PLR	platelet to lymphocyte ration
NLR	neutrophil to lymphocyte ratio
OS	overall survival
BMI	body mass index
HTN	hypertension
CVA	cerebrovascular accident
CEA	carcinoembryonic antigen
WBC	white blood cell
SHR	subdistribution hazard ratios
HR	hazard ratio
